# The revolution in pharmacotherapy: from herbs to pills, moulds, antibodies to genetic tools

**DOI:** 10.1093/ehjcvp/pvae098

**Published:** 2025-01-13

**Authors:** Thomas F Lüscher

**Affiliations:** Heart Division, Royal Brompton and Harefield Hospitals, GSTT, Sydney Street, London SW3 6NP, UK; King's College, Strand, London WC2R 2LS, UK; Center for Molecular Cardiology, Schlieren Campus, University of Zurich, Wagistreet 12, CH-8952 Schlieren, Switzerland

## From herbs to moulds

Medicine started with tender loving care, then with herbs such as foxglove as described by William Withering in 1784, and then moved to mould juice in 1928 when Alexander Fleming, at St George's Hospital, discovered that fungal mould of the strain *Penicillium rubens* inhibited bacterial growth and eventually led to the mass production of the powerful antibiotic penicillin during the closing years of World War II. In 1945, Howard Florey, Alexander Fleming, and Ernest Maurice Chain received the Nobel Prize for their discovery.^1^ When on 3 December 1967, Christiaan Barnard, performed the first heart transplantation, his patient, Louis Washkinsky, died just a few weeks’ later. Indeed, the importance organ rejection was massively underestimated by the pioneers. It required another fungus, i.e. *Tolypocladium inflatum*, to discover another breakthrough drug. Hartmann Staehelin noticed at Sandoz Pharmaceuticals in Basel, Switzerland, its potent, anti-inflammatory effects and eventually isolated the active compound cyclosporin, which revolutionized transplantation medicine.^2^ Another scientist, this time in Osaka, Japan, with the name of Akiro Endo, was inspired by the discovery of Fleming and speculated that some fungi, like moulds and mushrooms, might also inhibit 3-hydroxy-3-methyl-glutaryl-CoA reductase (HMG-CoA) enzyme A reductase, the rate-limiting enzyme for the formation of cholesterol. He began to use culture broths of fungi, and after 3800 strains tested, he found a culture broth of mould with potent inhibitory activity containing a known substance, citrinin, which eventually led to the first statins compactin and lovastatin.^3^ His discovery saved as many lives as Fleming's seminal observation.

## From fungi to antibodies

**Figure 1:**
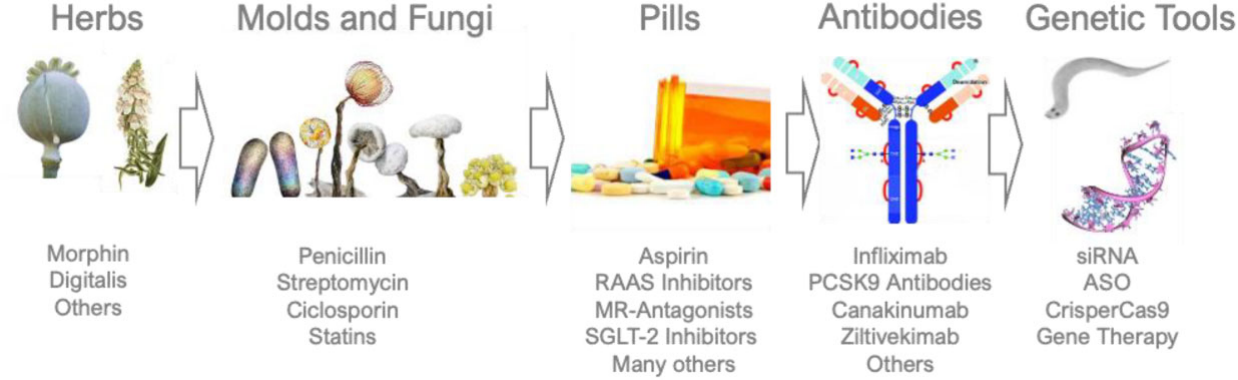
History of pharmacotherapy. Remedies developed from herbs to moulds, pills, antibodies and now to genetic tools revolutionizing pharmacotherapy further.

The race then continued from moulds to antibodies, taking advantage of the insights into the immune system and, in particular, of the antigen–antibody interaction. One of the first humanized monoclonal antibodies used in clinical practice was infliximab, a breakthrough for patients with rheumatoid arthritis. In cardiovascular medicine, the discovery of the innate immune system was instrumental, particularly the role of the inflammasomes, such as NOD-like receptor protein 3 (NLRP3), leading to the formation of interleukins and eventually C-reactive protein, the biomarker commonly measured in clinical practice.^4^ As pointed out by Rudolf Virchow in 1862, atherosclerosis was rediscovered as a chronic inflammation induced by cholesterol. A humanized monoclonal antibody against interleukin-1β, canakinumab indeed reduced major cardiovascular events (MACE) by around 15–20%,^5^ and similarly reduced the occurrence of lung cancer. Importantly, those in whom canakinumab supressed interleukin-6 most effectively had the lowest event rate. In line, the interleukin-6 antibody, ziltivekimab almost completely reduced plasma levels of C-reactive protein in patients after acute myocardial infarction^6^ and is currently tested in large randomized trials as to its capacity to reduce MACE. Concomitantly, scientists in Paris described a new mutation of the *PCSK9* gene causing autosomal dominant hypercholesterolaemia. Others discovered that there is not only a gain-of-function mutation but also a *loss-of-function* mutation associated with lifelong low LDL cholesterol levels and a massive 88% reduction in MACE in African American carriers.^7^ This led to a new class of PCSK9 inhibitors, injectable monoclonal humanized antibodies in hypercholesterolaemia (*Figure 1*).

## From antibodies to nucleoid acids

**Figure 2:**
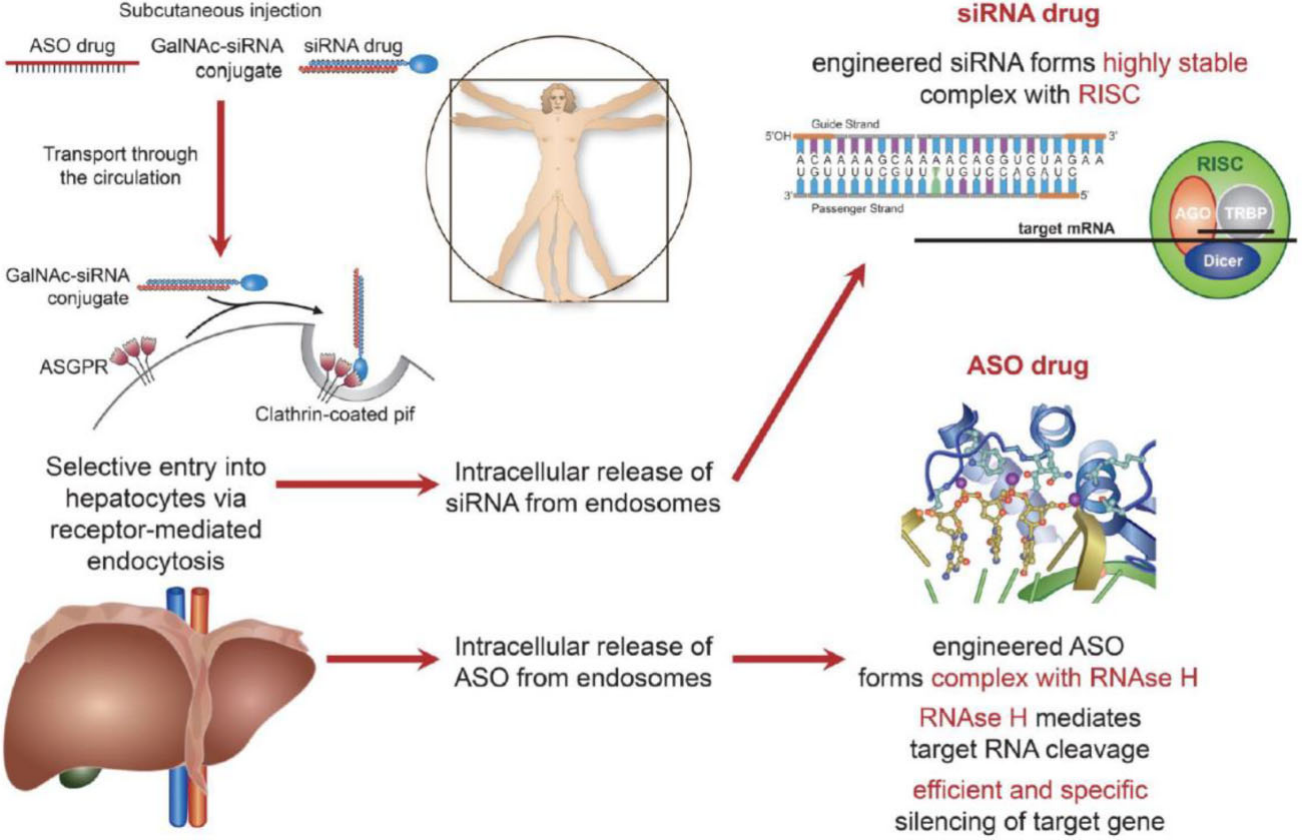
ASO and siRNA therapeutics. The liver has emerged as a reachable target, due to the delivery systems *N*-acetylgalactosamine (GalNac; ligand) binding to the asialoglycoprotein receptor (ASGPR) providing organ-specific siRNA and antisense oligonucleotide (ASO) application (from *Eur Heart J* 2020;41:3884–3899, by permission).

But, we moved further, from antibodies to nucleoid acids, i.e. genetic tools that interfere with the translation of the transcript to the protein in different cells.^8^ The seminal observation that double-stranded RNAs can bind to the RNA-induced silencing complex in the cytoplasm of cells and interfere with the translation of the RNA transcript to the mature protein was made by Andrew Z. Fire and Craig C. Mellow in the tiny worm Caenorhabditis  *elegans*. In 2006, they received the Nobel Prize in Physiology and Medicine for this seminal discovery that led to the development of therapeutic siRNAs in different medical conditions. For specific reasons, the liver is the main target of siRNAs, due to its expression of the asialoglycoprotein receptor (ASGPR) that is uniquely expressed in this organ and to which *N*-acetylgalactosamine (GalNac) residues coupled to double-stranded RNAs bind specifically. Using this principle, novel drugs evolved interfering with the production of PCSK9, to treat hypercholesterolaemia^9^ and, in turn, atherosclerosis and its

complications, to lower lipoprotein(a) for further risk reduction,^10^ to prevent the formation of angiotensinogen to lower blood pressure^11^ and eventually treat hypertension and potentially heart failure, as well as transthyretin (TTR) to reverse amyloid heart disease.^12^ Thus, these

new tools, unlike pills that have to be taken on a daily basis or antibodies to be injected on monthly or biweekly intervals, require only two to three injections a year, therefore massively reducing non-compliance with the prescribed treatment (*Figure 2*).

## From treatment to cure

Eventually, gene editing will require only one injection in a lifetime. Currently, this last revolution in pharmacotherapy was made possible by Emmanuel Charpentier and Jennifer Doudna, Nobel Prize awardees in 2006, with the development of CRISPR-Cas9, the gene scissor, allowing for editing of nucleoid acid sequences essential for the function of diverse genes and their proteins. Using this technology, Eric Olson's group was able to rescue dilated cardiomyopathy in the mouse with precise genomic editing of a pathogenic mutation in RBM20.^13^ In human embryos, Ma *et al*. successfully replaced a dysfunctional gene coding for hypertrophic cardiomyopathy with a wild-type variant.^14^ Finally, in TTR neuropathy, CRISPER-Cas9 has been successful in reducing TTR plasma levels once and forever in a small number of patients. These early observations opened the door for curative gene therapy of cardiomyopathies, which are currently only managed in a palliative fashion.

In primates, it was also possible using this technology to lower PCSK9 plasma levels by 90% and LDL-C plasma levels by 70%.^15^ Based on these experimental data, the US Federal Drug Administration gave clearance to an investigational new drug application VERVE-101, a CRISPER-Cas9 technology embedded into a microvesicle endowed with surface molecules binding to the LDL receptor on hepatocytes in patients with heterozygous familial hypercholesterolaemia. If safety and specificity of this new approach (*one shot in a lifetime*) can be demonstrated, CRISPR-Cas9 will open a completely new chapter in medicine and in cardiology in particular, allowing for cure rather than lifelong treatment with pills or antibodies.
